# Microneedles for *in situ* tissue regeneration

**DOI:** 10.1016/j.mtbio.2023.100579

**Published:** 2023-02-11

**Authors:** Linyu Long, Dan Ji, Cheng Hu, Li Yang, Shibo Tang, Yunbing Wang

**Affiliations:** a Aier Eye Institute, Changsha, Hunan Province, 410035, China; b Eye Center of Xiangya Hospital, Central South University, Changsha, 410008, Hunan, China; c National Engineering Research Center for Biomaterials, Sichuan University, Chengdu, 610064, China; d Aier School of Ophthalmology, Central South University, Changsha, Hunan, 410009, China; e CAS Center for Excellence in Brain Science and Intelligence Technology, Chinese Academy of Sciences, Shanghai, 200031, China

**Keywords:** Microneedles, Tissues regeneration, Drug delivery

## Abstract

Tissue injury is a common clinical problem, which may cause great burden on patients' life. It is important to develop functional scaffolds to promote tissue repair and regeneration. Due to their unique composition and structure, microneedles have attracted extensive attention in various tissues regeneration, including skin wound, corneal injury, myocardial infarction, endometrial injury, and spinal cord injury et al. Microneedles with micro-needle structure can effectively penetrate the barriers of necrotic tissue or biofilm, therefore improving the bioavailability of drugs. The use of microneedles to deliver bioactive molecules, mesenchymal stem cells, and growth factors *in situ* allows for targeted tissue and better spatial distribution. At the same time, microneedles can also provide mechanical support or directional traction for tissue, thus accelerating tissue repair. This review summarized the research progress of microneedles for *in situ* tissue regeneration over the past decade. At the same time, the shortcomings of existing researches, future research direction and clinical application prospect were also discussed.

## Graphical abstract

**Figure:**
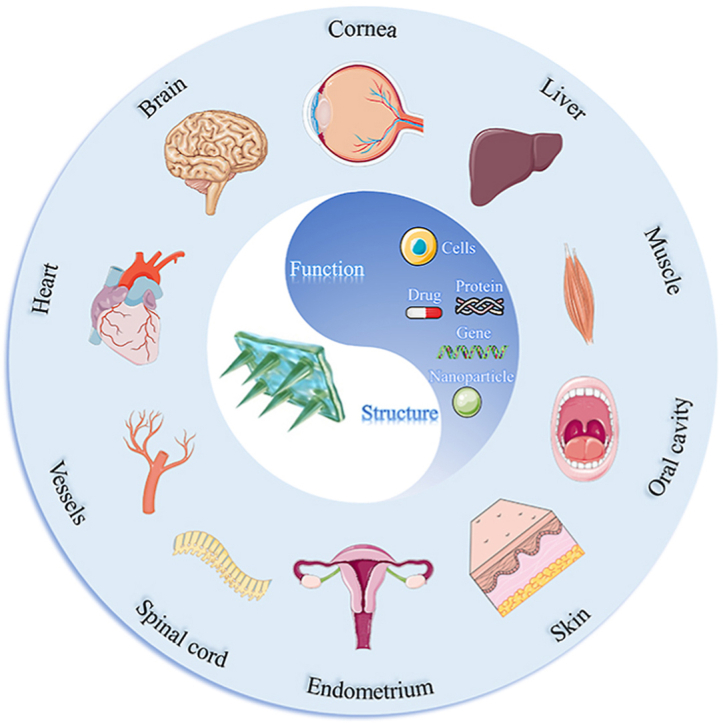

## 1 Introduction

Tissue repair or regeneration is one of the most complicated biological process, and refers to the restoration of damaged tissue to a normal state with full functions [[1], [2], [3]]. This process usually consists of three sequential stages of inflammation, tissue formation and maturation, and involves kinds of cells including immune cells, epithelial cells, endothelial cells [[4], [5], [6]]. Although small injuries can be healed through the body's adjustment, special regenerative therapy is usually required for non-self-healable pathological defects or large-scale damages [[7], [8], [9]]. Every year, millions of people die from damage or failure of organs or tissues worldwide, which prompts researchers to invest a lot of energy to explore advanced regenerative therapy [[10], [11], [12]]. Recently, with the in-depth exploration of tissue repair mechanisms and the development of biomaterials, the concept of *in situ* tissue regeneration (ISTR) has been introduced.

ISTR refers to the use of engineering biomaterials carrying biological active molecules (drugs, growth factors, genes, polypeptides, and cells, etc.) to direct endogenous progenitor or stem cells to repair damaged tissues [13,14]. Compared with *ex vivo* systems, ISTR is relatively convenient and eliminates the need for harvested cells, making it more suitable for clinical translation [15,16]. For example, NeuraGen and Neurotube for nerve repair, INFUSE bone grafts for dental or orthopedic applications [13]. Due to the complexity of the internal environment and tissue structure, biomaterials applied for ISTR should have comprehensive characteristics. For example, skin repair materials are required to possess tissue regeneration bioactivity, antibacterial effect and anti-inflammatory ability [[17], [18], [19]]. Angiogenesis is preferred for regeneration of vascularized tissues or organs such as heart and muscle [20], but should be inhibited during regeneration of avascular tissues such as cartilage and cornea [13,21]. To date, scaffolds including nanoparticles, fibers, hydrogels, microneedles (MNs), sponges and 3D printed scaffolds prepared from polymers, ceramics, metals and composites materials have been extensively investigated for ISTR [13,16]. Among them, MNs for minimally invasive delivery of therapeutic agents have become an emerging technology to promote tissue repair and healing, providing a novel development opportunity and challenge for ISTR.

The MN is a novel drug delivery device consisting of a substrate (area of mm^2^ to cm^2^) and micro-needles with a height of tens of microns to several nanometers [[22], [23], [24], [25], [26]]. Compared with traditional drug delivery methods such as injection and oral administration, MNs can painlessly penetrate tissue barriers [27], thereby improving the bioavailability of the drug and patients' adaptability, and avoiding the liver's over-mediated metabolism [13,28]. And MNs can also extract tissue interstitial fluid to obtain disease-related biomarkers [[29], [30], [31]]. As a result, MNs have been widely used to treat and detect various diseases, including skin cancer, infections, diabetes, obesity, and ocular diseases [[32], [33], [34], [35], [36], [37], [38]]. In the field of regenerative medicine, there are two main application pathways of MNs. One is transdermal delivery of active substances with MNs to promote tissue regeneration. For example, hyaluronic acid (HA) MNs to enhance transdermal uptake of exosomes for Achilles tendon repair [39], polyvinyl alcohol (PVA) MNs loaded with carbonized wormwood and inflammatory factors for skeletal muscles repair [40], and PVA MNs loaded with platelet derived growth factor for tendon healing [41]. Another is to use MNs as the scaffold for ISTR to enhance the healing ability of damaged tissues [13,42,43], which is also the focus of the present review.

**Fig. 1:**
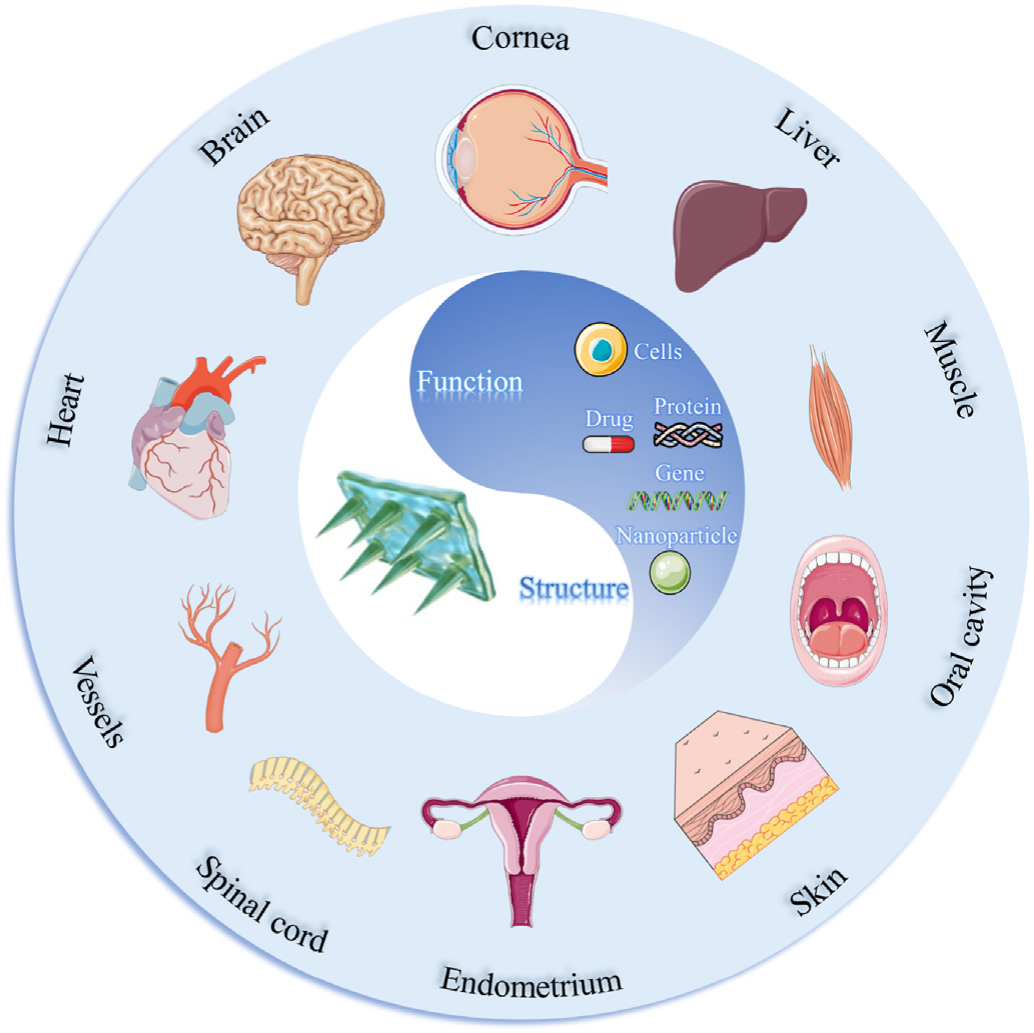
Schematic illustration of functional MNs for ISTR.

MNs can overcome necrotic tissue barriers or microbial membranes to deliver active substances minimally invasively to deep local tissues [44,45]. In addition, through the rational design of needle tips structure and basing, controlled release of active substances can be achieved conveniently and effectively. And MNs with special needle structures such as barbs can effectively close wounds and achieve rapid hemostasis or wound healing. Moreover, the basing of the MNs can act as sealant to prevent infection and tissue adhesions. In recent years, with the increasing understanding of the mechanisms of corneal healing, skin repair, nerve and heart regeneration and the rapid development of micro-/nano-technology, MNs-based ISTR has become a promising therapeutic strategy for tissue regeneration. MNs offer unique advantages in terms of improved patient convenience and drug bioavailability. And there are numerous reviews have discussed the preparation and classification of MNs and their applications in biomedical fields such as transdermal drug delivery, antibacterial, wound healing, and biosensing [42,44,46,47]. However, there are still few reviews that summarize and describe the principles and mechanisms of tissue repair using MNs. Herein, we systematically summarized and analyzed the major design concepts of MNs for ISTR and the applications of functional MNs in promoting the repair of damaged tissues such as skin wounds, corneal injuries, myocardial infarction (MI), and endometrial injuries (as shown in Fig. 1). And the shortcomings and clinical translation prospects of existing MNs-based tissue repair materials were also discussed.

## 2 Design principles of functional MNs for ISTR

### 2.1 Types of MNs

**Fig. 2:**
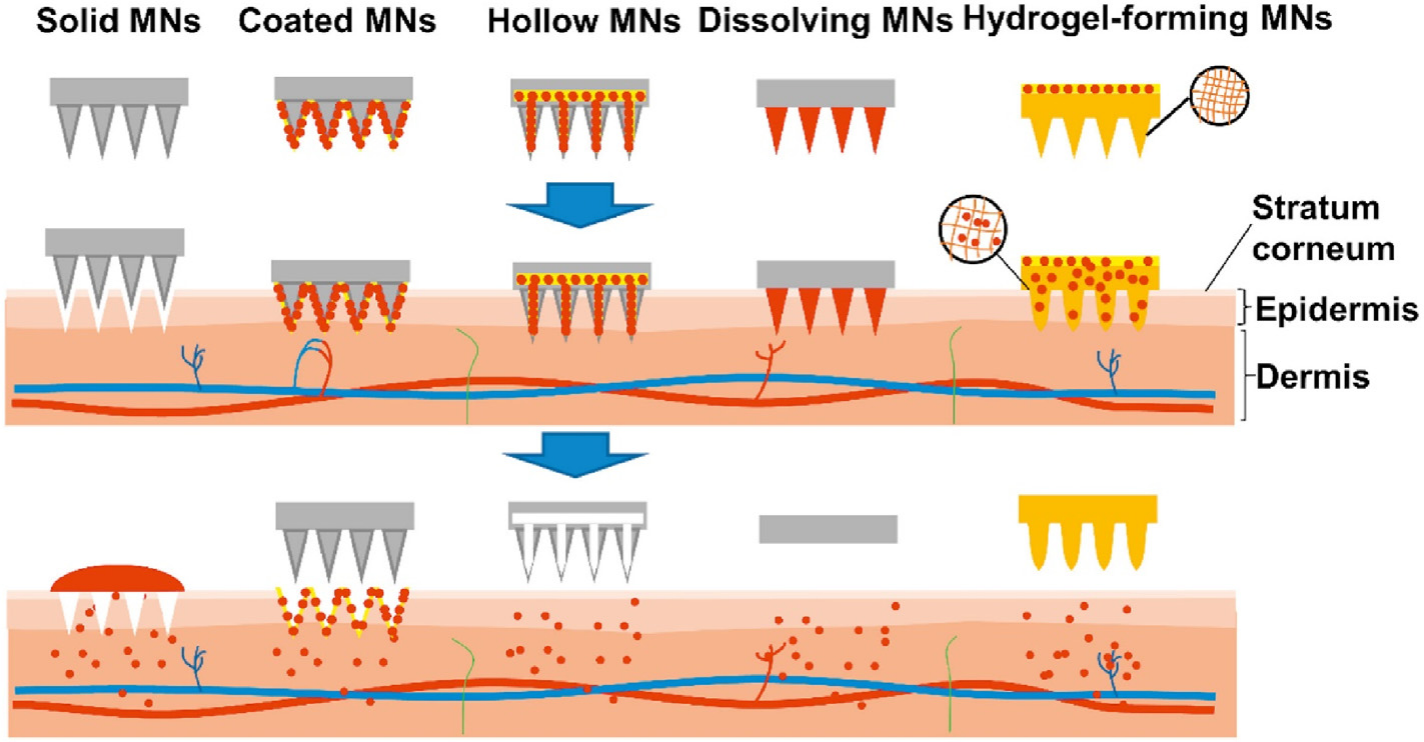
Representative five types of MNs (Reproduced with permission of Ref. [54]).

The first patent for MN was submitted by Gerstel and Place in 1976, but it was not until 1998 that the first paper on MN to enhance transdermal absorption of drugs was published [26,47]. Since then, the research on MNs has shown a spurt in development [48]. And the main methods for preparing MNs are: micromolding, mapping lithography, thermal drawing, electro-drawing, magnetorheological drawing lithography, additive manufacturing (3D printing) [42,[49], [50], [51], [52]]. Based on the mechanism of drug delivery, MNs can be classified as solid, hollow, coated, dissolving, and hydrogel-forming (as shown in Fig. 2) [53,54].

Solid MNs are mainly prepared from materials such as silicon, stainless steel or titanium and are commonly used as pretreatment strategies to form microchannels on the skin surface to improve drug diffusion efficiency [25,55,56]. However, poor biocompatibility, residual of sharp solid waste and uncontrollable drug dose limit their application in tissue regeneration [57,58]. The surface of coated MNs possesses drug coating formed by dip-coating or spraying methods, and the drugs are released after insertion [59]. The main disadvantages of coated MNs are the drug coating is easily wiped off during insertion and the drug loading capacity is insufficient [47,60]. Hollow MNs are usually used in combination with syringes, and the drugs are continuously delivered to deep tissues through a hole in the needle tip [54,59]. The main problems with hollow MNs are the risk of blockage or even blowout of the needle and the complexity of the preparation process [55,56]. Hollow and solid MNs can be viewed as miniature syringes that do not involve new drug dosage forms, so regulatory approval is more straightforward and quick. As a result, the U.S. National Food and Drug Administration (FDA) has approved two hollow MNs products for vaccination [49]. Currently, in the field of ISTR, hollow MNs and coated MNs are mainly used for corneal repair.

Dissolving, and hydrogel-forming MNs are the two types of MNs most frequently used in ISTR, due to their simple preparation process, flexible structural design or controlled drug release rate. Their common preparation technique is the solution-cast micromolding method, which uses a cavity prepared from polydimethylsiloxane (PDMS) as a master mold [42]. The materials used to prepare dissolving MNs are mainly dissolvable polymers (including HA, PVA, carboxymethyl cellulose (CMC), gelatin, sodium alginate (SA) and polyvinylpyrrolidone (PVP)) or biodegradable polymers (polycaprolactone (PCL), polylactic acid (PLA) and poly(lactic-co-glycolic acid) (PLGA)) [49,61]. After the dissolving MNs are inserted into the tissue, the drugs are released with the dissolution or degradation of the needle tip [55]. The selection of materials is critical for the properties of dissolving MNs such as mechanical strength, dissolution rate and tissue penetration. For example, compared with MNs prepared by SA, CMC and gelatin, HA MNs possess a faster dissolution rate, better mechanical strength and less shrinkage [62]. And MNs prepared from synthetic polymers such as PLGA and PLA typically have higher mechanical properties and slower drug release rates. The materials commonly used for the preparation of dissolving MNs and their advantages and disadvantages can be found in the published reviews [36,63].

Hydrogel MNs are made by cross-linking hydrophilic polymers such as poly(ethylene glycol) diacrylat (PEGDA) and gelatin methacrylamide (GelMA) [64,65]. The advantages and disadvantages of these materials can be found in the previously published reviews [66]. Hydrogels have high mechanical strength in the dry state and can penetrate tissues. After being inserted into the tissue, the MNs swell and then the drugs diffuse into the tissue [47,55,56,59]. Compared to dissolving MNs, hydrogel MNs have a slower and more controlled drug release rate [67]. In general, the mechanical strength of hydrogel MNs is directly proportional to the concentration and cross-linking time of the material, and the release rate of the drug tends to be inversely proportional [68].

MNs can painlessly and minimally invasively penetrate a variety of tissues, including skin and cornea, without causing pain or inflammatory response [69,70]. Compared to conventional injections or clinical implants, MNs allow controlled delivery of the active substance to the target site with minimal tissue damage, making them more suitable for self-administration by patients. Their multi-stage structure consisting of a needle tip and base greatly facilitates the design and regulation of the release rate of the active substance. When applied to ISTR, MNs are initially used only as a drug delivery tool. With the development of micro-/nano-technology and better understanding of tissue repair mechanisms, different functional MNs are designed. Currently, the main roles of MNs in the tissue repair process are: 1. enhancing the delivery efficiency of active substances to improve bioavailability, 2. regulating the release rate of multiple active substances to achieve on-demand release, and 3. providing mechanical support or physical stimuli to regulate cellular behavior for rapid wound healing.

### 2.2 Enhancing local delivery efficiency

**Fig. 3:**
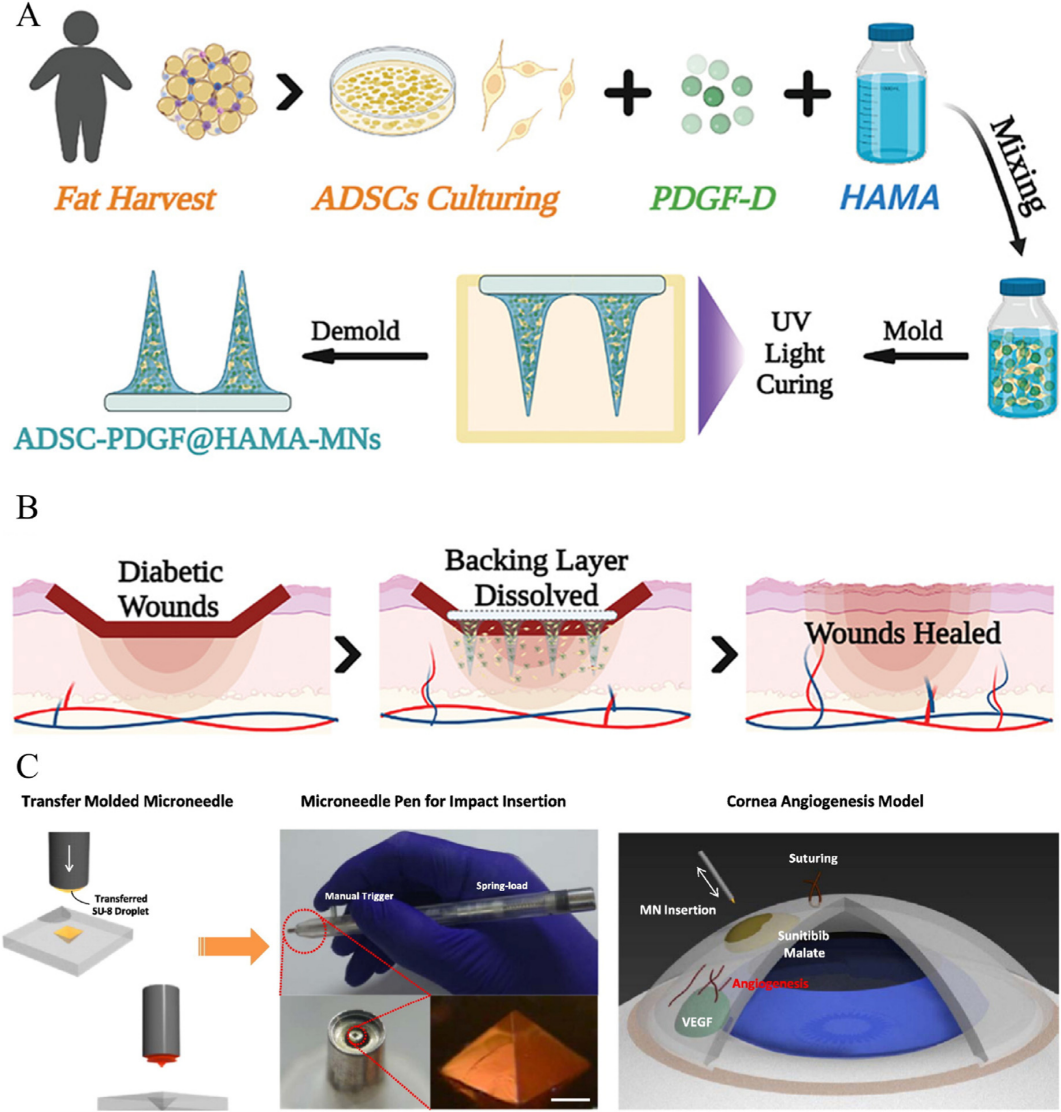
A) Schematic illustrations of the MNs loaded with ADSCs and PDGF-D. B) Application of MNs for diabetic wound treatment (Reproduced with permission of Ref. [72]). C) Schematic diagram of transfer-molded MN for localized and minimally invasive ocular drug delivery (Reproduced with permission of Ref. [77]).

Due to the lack of penetration ability of conventional scaffolds such as hydrogels and sponges, necrotic tissue barriers or biofilms at damaged tissues may limit the permeability of active substances and lead to low bioavailability and poor therapeutic efficacy. Here, MNs with micro-needles structure provide an effective solution without using syringes which may cause inflammation and pain. For instance, by means of simple finger pressure, MNs can penetrate the skin tissue and deliver the active substance to the wound bed [71]. And MNs can effectively encapsulate cells or bioactive substances and maintain their good activity. As shown in Fig. 3A and B, Prof. Zhao's group fabricated methacrylated HA (HAMA) hydrogel MNs encapsulated with bioactive platelet-derived growth factor D (PDGF-D) and human adipose stem cells (ADSCs) [72]. The HAMA MNs could maintain the activity of ADSCs over 90% for 24 ​h, enhance the biological function of ADSCs to secret growth factor and promote diabetic wound healing by accelerating vascularization, tissue regeneration and collagen deposition. In addition, mature biofilms form a protective microenvironment to hinder the penetration of antimicrobial substances, while MNs can effectively penetrate biofilms [29,[73], [74], [75], [76]]. However, for MNs, effective drug delivery to thin tissues with little background support (e.g., the cornea) has been a challenge. As shown in Fig. 3C, Ryu et al. designed a pen system with a spring-loaded applicator to provide impact force to insert a single MN into the corneal tissue [77]. This MN system allowed for a more localized and minimally invasive delivery of sunitinib to the target site compared to subcutaneous needles. In a suture-induced corneal angiogenesis model, the MN system effectively inhibited corneal neovascularization *in vivo*.

By utilizing the microenvironment of damaged tissue sites (e.g., enzymes, pH) or *in vitro* stimulus such as near-infrared (NIR), intelligent MNs can be designed to control the release rate of active substances. Xia et al. encapsulated gelatin nanoparticles modified with the gelatinase-responsive antimicrobial photothermal peptide AMP-Cypate in dissolving MNs [78]. When applied to the infected site, the MNs broke through the epidermis and stratum corneum and released the nanoparticles after dissolving. And the nanoparticles could release AMP-Cypate with photothermal antimicrobial properties in response to gelatinase secreted by *S. aureus*. Similarly, Wang et al. prepared NIR photothermally responsive NO-releasing MNs by embedding graphene oxide particles loaded with nitric oxide and metal organic frameworks into the porous structure of PEGDA hydrogel MNs [79]. Zhao et al. embedded black phosphorus quantum dots and oxygen-loaded hemoglobin into GelMA hydrogel MNs [80]. Upon NIR irradiation, the MNs could responsively release oxygen to treat diabetic wounds.

It must be noted that due to the variability of mechanical strength and thickness of different tissues, the physical properties required for MNs to effectively deliver the active substance to the target tissue are different. For the design and preparation of MNs for tissue regeneration, the dimensions of the needles of MNs (including shape, height, area of the needle tip, and mechanical strength, etc.) are the first things worth considering. Due to the good tissue penetration of the conical structure [81], the needle shapes of MNs currently used for tissue regeneration are mainly conical or pyramidal (quadrangular conical). The mechanical strength of MNs is mainly related to the shape of MNs and the area or materials of the needle tip [82]. Studies have shown that the strength of MNs exceeding 0.05 ​N/needle can penetrate tissues such as skin, cornea, endometrium and heart [[83], [84], [85]], while most of the current MNs have a strength exceeding 0.1 ​N/needle and are sufficient for use.

Overall, recent researches have demonstrated the spatial precision of MNs for drug delivery while overcoming the complex and dynamic barriers of various tissues including eye and skin [86]. Clinical studies of regenerative therapies based on active substances such as stem cells and growth factors have been stalled by their susceptibility to inactivation and low transplantation efficiency, and MNs may offer a promising solution for them. MNs with unique micro-needle structures can integrate bioactive materials and host tissues well, thus improving the contact area and depth of the biomaterial with the tissue, and enabling better regulation of cellular behavior to promote damaged tissue repair. However, parameters such as material, height, number and mechanical strength of tips should be fully considered when designing and preparing MNs for different damaged tissue repair.

### 2.3 Regulating the release of multiple active substances

Due to the complexity of the tissue environment, additional demands are placed on the functions of MNs used for tissue repair. For example, MNs for bacterial infected wound repair require antimicrobial properties, pro-angiogenesis and tissue regenerative properties, and MNs for MI repair require pro-angiogenic activity and inhibition of myocardial fibrosis.

Simply, multifunctional MNs can be obtained by incorporating multiple active substances into polymer solutions using a micromolding method. By this method, Professor Wang's group prepared HA MNs co-loaded with recombinant humanized type III collagen (rhCol III) and naproxen (Nap) encapsulated PLGA nanoparticles [87]. After being inserted into the wound bed, the prepared MNs would rapidly dissolve within few minutes, thereby releasing rhCol III and PLGA nanoparticles. And PLGA nanoparticles could slowly release Nap (release of more than 70% of the drug over 14 ​d) in the dermis to effectively inhibit the inflammatory response in the wound site. The side effects caused by the explosive release of dissolved MNs can be better avoided by using the slow-release properties of nanoparticles. In another research, with the dissolution of MNs, ovofloxacin directly doped in PVP MNs was almost completely released within 15 ​min, whereas protein drugs encapsulated in PLGA microspheres were consistently released for a long time (more than 86% of the drug over 15 ​d) [88]. The advantage of this design was that the rapid release of antibiotics effectively inhibited bacterial infection, while the sustained release of bFGF promoted tissue regeneration throughout the repair process.

**Fig. 4:**
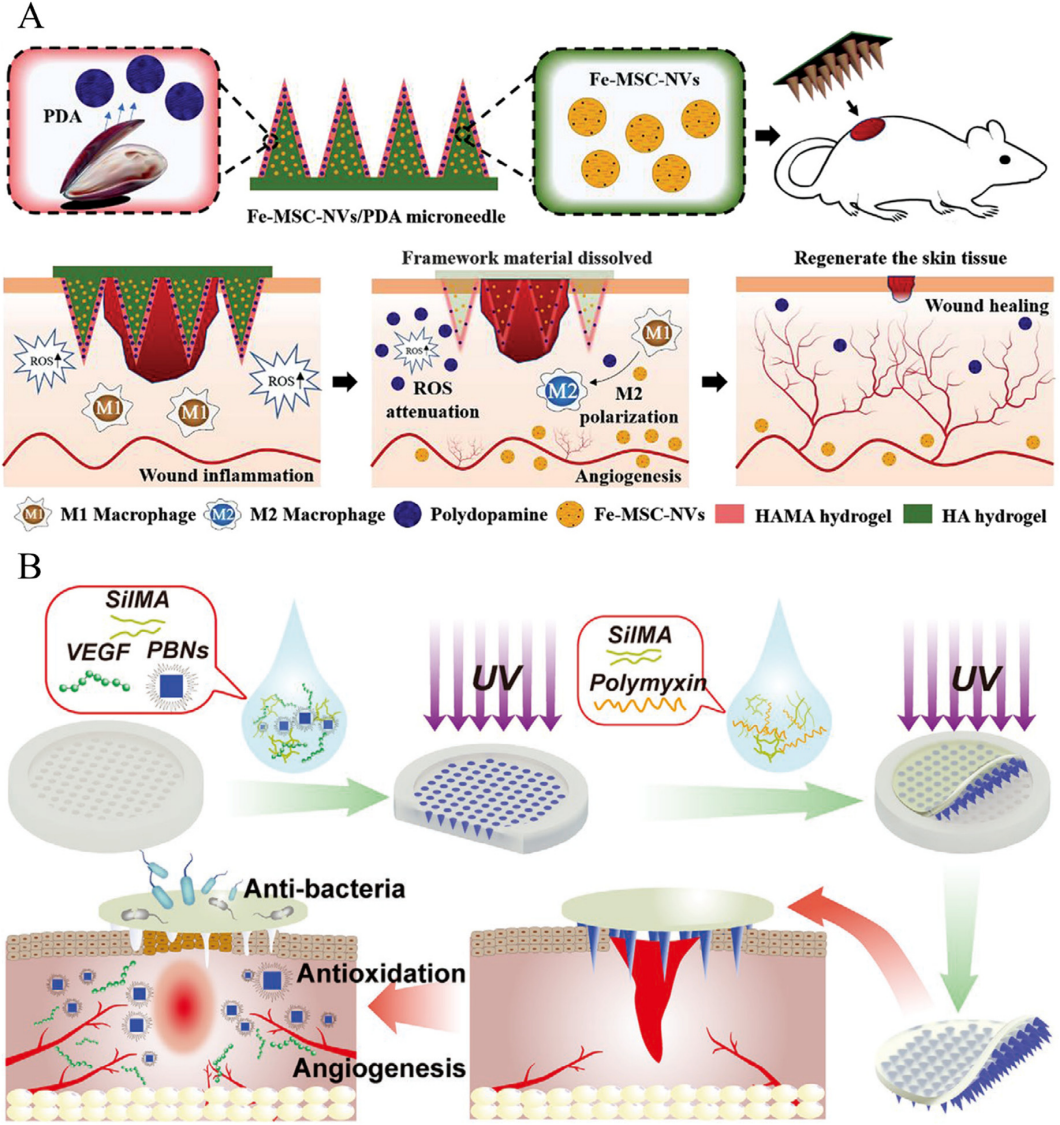
A) Schematic illustrations of Fe-MSC-NVs/PDA MN for diabetic wound healing (Reproduced with permission of Ref. [89]). B) Schematic presentation of MN-PBNs-VEGF for diabetic wound healing (Reproduced with permission of Ref. [93]).

The second route is to apply the concept of coated MNs and prepare multilayer composite MNs to regulate the release rate of drugs. For example, as illustrated in Fig. 4A, ferrum-mesenchymal stem cell-derived artificial nanovesicles (Fe-MSC-NVs) with pro-angiogenic activity were encapsulated in the internal HA core of the MN tip, while polydopamine (PDA) with antioxidant function was encapsulated in the HAMA hydrogel shell [89]. With the rapid dissolution of HA, more than 95% of Fe-MSC-NVs were released within 5 ​h; whereas for PDA in HAMA hydrogels, only about 80% was released after 70 ​h. PDMS molds for the preparation of such bilayer MNs required plasma treatment to enable polymers with hydrophilic and adhesive properties to adhere to the mold surface, ultimately forming a stable and robust shell [90]. The above two methods achieve the release of multiple active substances through the MNs tips, however, the loading amount raises concerns due to the small size of the tip.

Another approach is to embed other active substances in the base of MNs at the same time, which can effectively provide programmed treatment to damaged tissues [91]. In addition, this type of MN allows better control of the spatial distribution of the drugs in the target tissue, an advantage that other types of materials such as hydrogels or sponges do not have. For example, antimicrobial materials in the base of MNs can effectively remove bacteria from the wound surface and prevent infection, while pro-repair materials at the needle tips can be delivered to the dermis for enhanced bioactivity and retention [92]. As shown in Fig. 4B, the prussian blue nanoenzyme (PBN) and vascular endothelial growth factor (VEGF) were encapsulated in the tips, while polymyxin was encapsulated in the base of silk fibroin methacryloyl (SilMA) MNs. The base of MNs could release polymyxin to inhibit bacterial infection. Due to the interconnected porous structure of hydrogel MNs, PBN and VEGF could be slowly released into the wound tissue after the MNs swelling [93]. The use of the base, which plays a mechanically supportive role, as a drug carrier allows the preparation of multifunctionally integrated MNs and can, to a certain extent, solve the problem of low loading of dissolving and hydrogel MNs.

Interestingly, when the needles and base of the MNs are made of materials with different dissolution or degradation rates, the drug release rate can be flexibly controlled. For example, a bilayer MN consisted of tetracycline hydrochloride (TCH) loaded HA needles, and deferoxamine (DFO) loaded chitosan and silk fibroin base. The TCH was completely released from HA needles in 25 ​min to promote early antimicrobial activity, while the release of DFO in the base was relatively slower [94]. It is worth mentioning that the interface between the needles and the base may have an impact on the release rate and delivery efficiency of the drug. For example, the addition of air bubbles or materials that dissolve quickly at the union of the two layers can enable rapid separation or push the needles into deeper tissue [91].

For dissolving MNs, encapsulating drugs in materials such as nanoparticles, micelles, and hydrogels that can release drugs continuously and slowly can better circumvent the negative effects of their explosive drug release and control the release rate of multiple drug combinations. Bilayer MNs prepared from a combination of dissolvable and hydrogel materials are an important research direction for the future. This is because they allow for increased drug loading and ease of use, while also intelligently controlling the rate of drug release according to the requirements of tissue repair process. Compared to direct doping method, bilayer MNs offer more flexible active substance combinations and easier control of release rates. However, the preparation process of bilayer MNs is more tedious and the reproducibility of the product may be less demanding. Considering the sequential nature of the tissue regeneration process, the on-demand release of active substances is particularly important. However, the preparation of MNs with drug release rates that can perfectly match the tissue healing process is still a considerable challenge due to the limitations of MNs preparation technology.

### 2.4 Providing mechanical support or physical adjustment

In addition to acting as a carrier of active substances, MNs, a minimally invasive medical device, can also modulate cellular behavior and provide a favorable environment for tissue repair. Luo et al. found silk fibroin (SF) MNs induced impairment of mechanical communication between fibroblasts and ECM, as well as reduced mechanical stress and contraction generated by fibroblasts [95]. In addition, SF MNs attenuated integrin-FAK signaling, thereby downregulating the expression of TGF-β1, α-SMA, collagen I, and fibronectin. However, although this report investigated the therapeutic effects of three different sizes of MNs (arrays of 5 ​× ​5, 10 ​× ​10, and 15 ​× ​15 with heights of 500, 1000, and 1500 ​μm), it lacked consideration of the physicochemical properties such as the shape and mechanical strength of MNs. In conclusion, as a minimally invasive option, the SF MNs had great potential to provide cost-effective and convenient therapy for scar-free wound healing. For the treatment of MI, MNs can also act as myocardial patches to reduce wall stress and strain in the infarcted area and maintain left ventricular function and morphology [96].

**Fig. 5:**
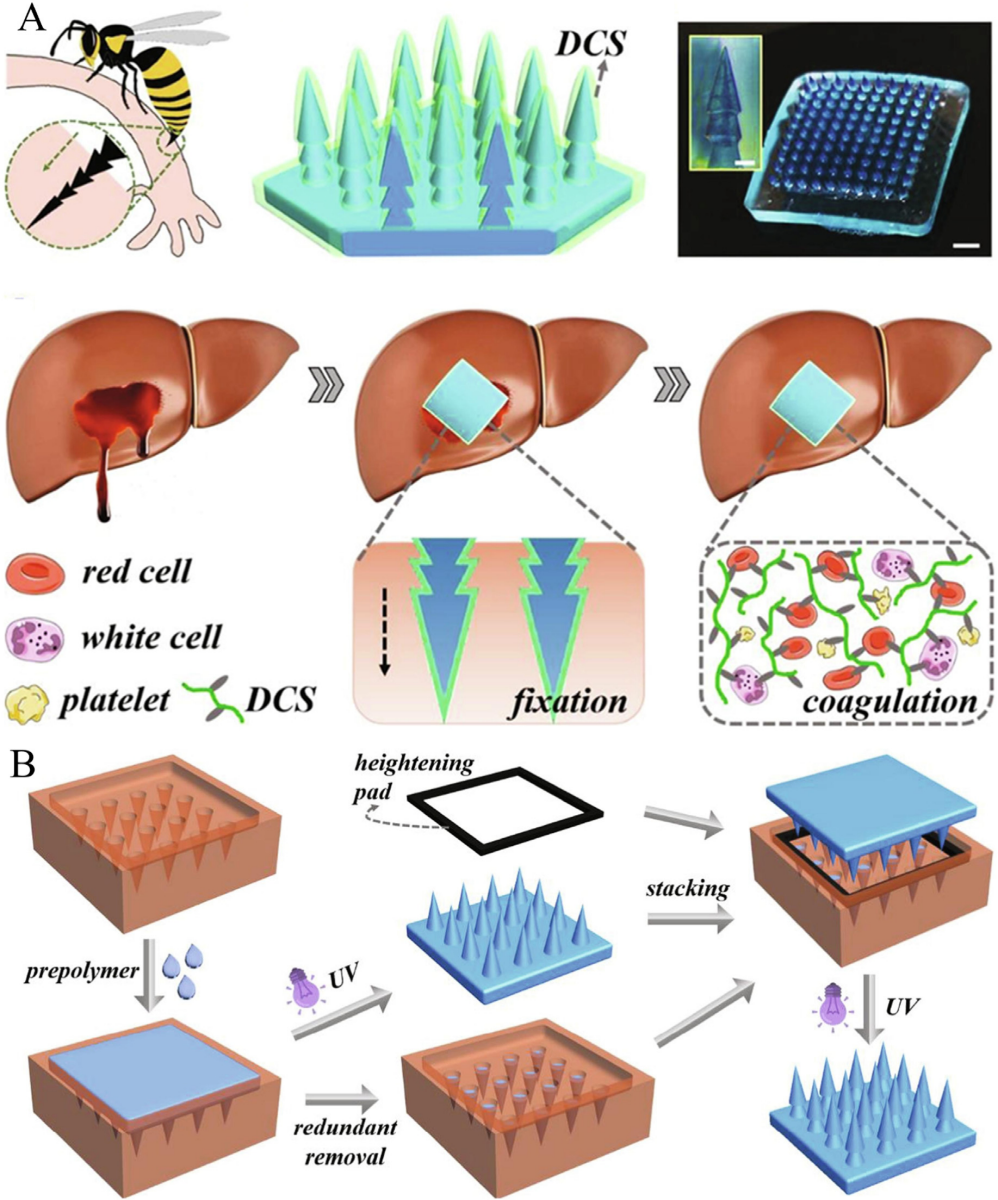
A) Design and hemostatic function of the bioinspired pagoda-like MNs. B) Schematic illustration of the fabrication process (Reproduced with permission of Ref. [98]).

Mimicking nature's layered architecture and sophisticated strategies, bionic MNs with special structures are designed and manufactured to adhere tightly to tissue surfaces or generate traction to close wounds [42]. For example, inspired by the expandable proboscis of endoparasites, Cha et al. developed a bilayer adhesive hydrogel MN consisting of an expandable mussel adhesion protein shell and a non-expandable SF core [97]. The prepared MNs could replace sutures for rapid closure of gastrointestinal/skin wounds and release pro-regenerative or anti-inflammatory agents to the target tissue. Inspired by the layered microstructure of foot or insect bites, Prof. Zhao's group prepared a dodecyl-modified chitosan (DCS) coated pagoda-like multilayer hydrogel MNs by repeatedly stacking MN and molds (as shown in Fig. 5) [98]. The prepared MNs exhibited excellent hemostatic properties in a rabbit model of acute tissue injury (e.g. liver hemorrhage, spleen hemorrhage and kidney hemorrhage). Similarly, other bionic MNs inspired by lamprey-teeth [99], claws of eagles [100], and shark tooth [101], had been designed and manufactured to produce traction to close wounds and promote wound healing. However, the preparation of bionic MNs often requires complex processes or places higher demands on the precision of the equipment, which may be a major obstacle to their clinical translation and industrial production.

In general, relevant studies have shown that MNs can provide physical stimulation, traction, and support to repair damaged tissues. However, the specific therapeutic mechanism remains to be further demonstrated. To date, most studies on MNs have focused on MNs as minimally invasive devices for drug delivery, and few have investigated the effects of the structure or materials of MNs on tissue repair. Surprisingly, with the development of micro and nano processing technology, some bionic MNs with specific structures have been designed and prepared for ISTR. These MNs have shown promising therapeutic effects and potential in areas such as wound closure, myocardial repair and hemostasis of the liver. However, the complexity of their preparation process also poses a challenge for their further research and development.

## 3 MNs for ISTR

### 3.1 MNs for skin repair

Skin wound care is a major social and economic burden, with millions of people suffering from poor wound healing each year worldwide. Approximately 1–2% of the population in developed countries is reported to suffer from chronic wounds [37]. The normal skin wound healing process includes hemostasis, inflammation, proliferation and tissue remodeling, involving various types of cells and bioactive factors [44]. Diseases such as diabetes, nasty skin tumors or infections can delay wound healing and eventually result in the development of chronic wounds [102]. In chronic wounds, the presence of scabs and microbial membranes, exudate drainage, and a hostile chemical microenvironment rich in various enzymes can undermine the therapeutic efficacy of topical drug delivery [103]. Recent studies have shown that MNs can painlessly puncture the epidermis to form microscopic pores which facilitate the rapid diffusion of biologically active substances (drugs, cells, growth factors, genes, etc.) to the wound bed [43,46,[104], [105], [106]]. Table 1 summarizes representative recent studies of MNs for skin tissue regeneration. As we well known, the total thickness of the dermis and stratum corneum of the skin is about 200 ​μm, and the length of MNs for skin tissue regeneration is typically 500–1000 ​μm [26], taking into account the compressive deformation of MNs and the elasticity of the skin.

**Table 1:** MNs for skin tissue regeneration.

Materials	Structure	Bioactive substances	Mechanisms	Ref.
Base: gelatin Needles: HAMA	Arrays: - Height: 800 ​μm Shape: conical Strength: Failure force about 2 ​N	Platelet derived growth factor D, human adipose-derived stem cells	Ensuring sufficient nutrient supply to sustain viability of cells >90% for 24 ​h.	[72]
Needles: GelMA Base: polyvinyl acetate	Arrays:- Height:- Shape: conical Strength: >0.5 ​N/needle	Oxygen	The base would soon disappear by dissolving within 10 ​min; Using the photothermal effect of the black phosphorus and the reversible oxygen-binding property of hemoglobin, the needle tip released oxygen under NIR irradiation.	[80]
Needle: outer PLGA shell and internal GelMA Base: doublesided tape	Arrays:- Height: 700 ​μm Shape: conical Strength: Failure force of 1–1.5 ​N	Mesenchymal stem cells	>90% of MNs fall off over 1 ​min; Cell viability remained above 90% up to 24 ​h in MNs; Delivery of cell-secreted VEGF.	[86]
HA	Arrays: 10 ​× ​10 Height: 600 ​μm Shape: pyramidal Strength: 3.98–5.72 ​N	Recombinant humanized collagen type III, naproxen	Drugs were rapidly released to the wound site within a few minutes.	[87]
PVP	Arrays: 10 ​× ​10 Height: 530 ​μm Shape: conical Strength: 3.98–5.72 ​N	bFGF, ofloxacin	Ofloxacin was quickly released to inhibit infection due to the fast dissolution of MN; bFGF loaded in PLGA microspheres was slowly released to further promote wound healing.	[88]
HA, HAMA	Arrays: 20 ​× ​20 Height: 860 ​μm Shape: pyramidal Strength:	PDA nanoparticles, Fe-MSC-NVs	Fe-MSC-NVs loaded inner HA core of the MN tips accelerated angiogenesis; PDA nanoparticles in the outer HAMA shell could be sustainably released at the lesion to suppress the ROS	[89]
Base: PVP Needles: chitosan	Arrays: 20 ​× ​20 Height:- Shape: conical Strength: >0.25 ​N/needle	Magnesium, Panax notoginseng saponins	The back dissolved within 9 ​min to release Mg^2+^; The hydrogel needles slowly released the drug into the dermis.	[91]
Poly(γ-glutamic acid)	Arrays: 10 ​× ​10 Height: 500 ​μm Shape: pyramidal Strength:-	Magnesium MOF, graphene oxide-silver nanocomposites	The base inhibited bacterial infection; The needles slowly released Mg^2+^ and gallic acid in the deep layer of the dermis.	[92]
Silk fibroin methacryloyl	Arrays: 385 needles Height: 700 ​μm Shape: conical Strength:-	Prussian blue nanozymes, VEGF, polymyxin	Polymyxin in the base inhibited bacterial infections; Nanozymes and VEGF loaded needles facilitated diabetic wound healing.	[93]
PVA	Arrays: 10 ​× ​10 Height: 750 ​μm Shape: pyramidal Strength:-	α-amylase, levofloxacin-loaded polydopamine nanoparticles	The needle tip penetrated the biofilm; Under NIR irradiation, amylase was released to expose bacteria in the biofilm and combine the photothermal effect and antibiotics to kill the bacteria.	[107]
Chitosan	Arrays: 20 ​× ​20 Height: 600 ​μm Shape: conical Strength:-	VEGF	VEGF loaded temperature responsive hydrogel in needles released drugs induced by the inflammation response at the site of wounds.	[113]
Poly(γ-glutamic acid)	Arrays: 10 ​× ​10 Height: 500 ​μm Shape: pyramidal Strength: >1 ​N/needles	MXenes, asiaticoside	MXenes improved the mechanical strength and reduced the drug release rate of MNs; Penetrating cuticle for subcutaneous drug delivery.	[115]
GelMA, 4-(2-acrylamidoethylcarbamoyl)-3-fluorophenylboronic acid	Arrays: 11 ​× ​11 Height: 600 ​μm Shape: conical Strength:-	Insulin	Glucose-responsive insulin release to control glucose in diabetic patients.	[117]
Needles: GelMA Base: silk fibroin methacryloyl	Arrays: 20 ​× ​20 Height: 600 ​μm Shape: conical Strength:-	AgNPs, mesenchymal stem cell- derived exosomes,	Adhering steadily to the skin around the wound edges to form an anti-infective surface; Delivering exosomes to the dermis.	[119]
Base: alginate, gelatin, HA Needles: HA	Arrays: 20 ​× ​20 Height: 540 ​μm Shape: pyramidal Strength: >0.1 ​N/needle	Curcumin, Indocyanine Green	The needles would rapidly dissolve and release drugs to remove tumor under NIR irradiation. Hydrogel base covered the wound and promoted the cells proliferation.	[121]
Silk fibroin, silicon	Arrays:- Height:- Shape: conical Strength: 0.5 ​N/Needle	VEGF	Porous structures enhanced the drug loading capability; Liquid diffused along the microfluidic channel for detecting biomarkers in wound secretion; Microelectronic circuits were integrated to sense the motion state of wound areas.	[123]
HA	Arrays:- Height: 600 ​μm Shape: conical Strength:	Platinum–ruthenium nanoalloys and porous graphitic carbon nitride C3N5 nanosheets	Improving transdermal drug delivery efficiency	[124]

Bacterial infected wounds have become a major global healthcare problem, with 60–90% of hard-to-heal wounds associated with biofilms [107]. Fortunately, antimicrobial MNs have been shown to provide effective treatment for infected wounds. The most common method of preparing antimicrobial MNs is to encapsulate antimicrobial agents in the tip or backing of MNs. For example, Yan et al. incorporated the photosensitizer Zn_2_GeO_4_:Cu^2+^ (ZGC) into dissolving MNs [108]. The prepared MNs penetrated biofilms in a minimally invasive manner and delivered pre-UV-treated ZGC to infected wounds. The ZGC could produce multiple reactive oxygen species (ROS) (^1^O_2_, hydroxyl radicals and superoxide radicals) continuously over 48 ​h, effectively eliminate methicillin-resistant *Staphylococcus aureus* (MRSA) biofilms, reduce *in vivo* inflammation and promote wound healing. Similarly, antimicrobial agents such as water-soluble antibiotics (ofloxacin [88], asiatic acid [109]) or antimicrobial nanomaterials (Metal organic framework (MOF) [110], silver nanoparticles (AgNPs) [111], and nanozyme [112]) have been successfully encapsulated into MNs and demonstrated excellent antimicrobial properties and the ability to promote infected skin wounds regeneration. Another way to prepare antimicrobial MNs is to use antimicrobial polymers such as chitosan as the main material [113,114]. Zhao et al. fabricated an antibacterial and angiogenic chitosan hydrogel MNs by encapsulating the VEGF-loaded temperature-sensitive hydrogels into the porous network of needles [113]. The prepared MNs could release VEGF induced by the rise of the body surface temperature attributing to the inflammation response.

**Fig. 6:**
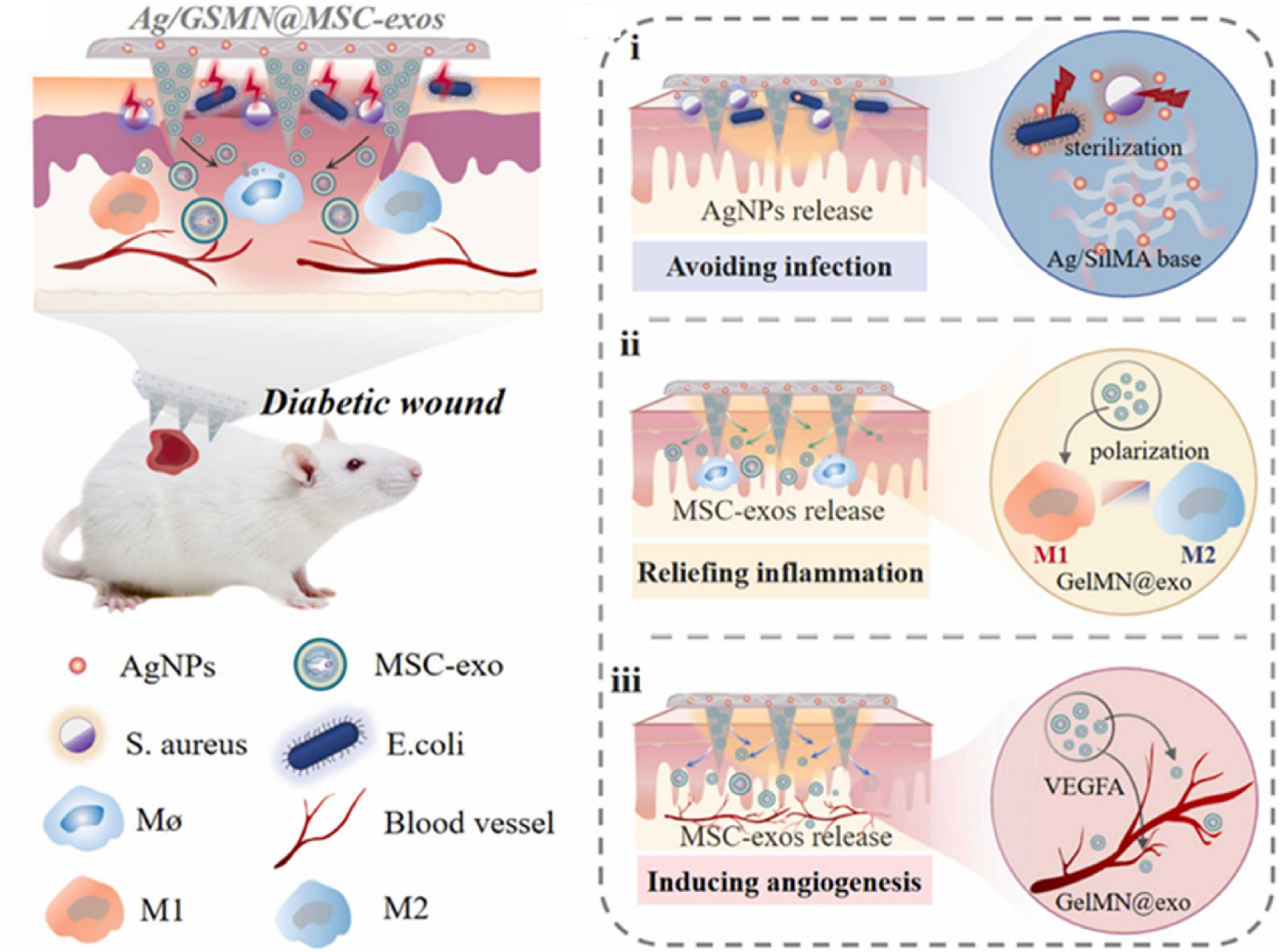
Illustration of the MSC-exos loaded MN patch for promoting wound healing (Reproduced with permission of Ref. [119]).

The global prevalence of adult diabetes is expected to increase to 7.7% by 2030, and diabetic complications pose a serious threat to patients' health, leading to reduced quality of life, increased healthcare costs and mortality [64,115]. In particular, approximately 15–25% of diabetes will develop a diabetic foot ulcer (DFU) during their lifetime [92,116]. DFU is often accompanied by persistent inflammation, bacterial infection, poor angiogenesis and tissue regeneration, making it often difficult to cure DFU with traditional treatment [117,118]. As illustrated in Fig. 6, Zhao et al. prepared an adherent MNs encapsulated with mesenchymal stem cell-derived exosomes (MSC-exos) and AgNPs for improving diabetic wound healing [119]. The tips of the MNs consist of GelMA hydrogel that could continuously release anti-inflammatory and pro-angiogenic MSC-exos to the wound bed, therefore accelerating healing by improving cell function, remodeling blood vessels and restoring the immune system. In addition, the AgNPs loaded backing composed of SF allowed MNs to adhere well to the skin and anchor to the wound site.

MNs can effectively penetrate the skin, increase drug distribution to deeper tumor sites, and minimize leakage of therapeutic drugs to adjacent tissues, thus improving the therapeutic effect of cutaneous epidermal tumors [120]. On the other hand, MNs with tissue repair function can simultaneously promote skin wound healing after tumor eradication [121]. Yu et al. prepared HA MNs functionalized with silica-coated melanin nanoparticles for simultaneous tumor photothermal therapy (PTT) and promotion of skin tissue regeneration [122]. The prepared MNs could remove residual subcutaneous tumor cells and inhibit bacterial infection in the wound bed by PTT. In addition, they could also modulate inflammatory responses, upregulate angiogenic gene expression and promote skin tissue regeneration by scavenging ROS and releasing proto-silicate ions.

In conclusion, MNs can provide effective treatment for difficult-to-heal wounds such as bacterially infected wounds, diabetic wounds, and wounds caused by tumor resection. Considering the complexity of the wound physiological environment, bilayer MNs with flexible drug combinations and controlled drug release rates are one of the hot spots for future research. However, it is important to consider that the thickness of the skin layer varies from patient to patient, making the penetration depth of MNs potentially very different, leading to a wide range of abnormalities in the spatial distribution of active ingredients. Furthermore, most MNs for tissue regeneration are prepared by using fixed-shape molds. Despite the simplicity and reproducibility of the preparation process, also makes it difficult to provide effective treatment of irregular wounds with MNs. Although the shape and size of the mold can be personalized using image recognition [125], it also increases the difficulty of the process. And for acute wounds or burns what the dermis is exposed, minimally invasive MNs may damage capillaries and lead to increased inflammation. Finally, porous MNs such as hydrogel MNs can aspirate biomarker-rich skin interstitial fluid and can monitor tissue healing during treatment [123,126], but their sensitivity and timeliness still need to be improved.

### 3.2 MNs for corneal regeneration

The cornea, a multi-layered transparent tissue without blood vessels, is the most densely distributed nerve tissue in the body, with approximately 7000 injurious receptors per square millimeter [[127], [128], [129]]. Corneal damage is the second leading cause of blindness in developing countries, and approximately 10 million patients worldwide have been diagnosed with bilateral corneal blindness [130]. Despite genes, stem cells, gels and drops have been developed, human donor corneal transplant is the most effective treatment for corneal blindness and restoring patients' vision (with a cure rate of more than 80%) over a century. In 116 countries, approximately 185,000 corneal transplants are performed each year, yet 13 million people are still waiting for corneal transplants [[130], [131], [132]]. Therefore, there is an urgent need to find novel strategy to promote corneal regeneration. The micron-sized MNs can overcome the disadvantages (frequent, invasive and low bioavailability) of traditional injections, and have been widely used for the treatment of ocular diseases such as corneal injury, glaucoma, age-related macular degeneration and uveitis [30,[133], [134], [135], [136]]. MNs have been used for the treatment of ocular diseases over 10 years, and there are still no reports of serious side effects of MN injections on eye tissue.

Table 2 summarizes representative recent studies of MNs for corneal regeneration. Considering the cornea has a certain curvature (39–45 D), MNs for corneal repair usually have a small number of needles. It is worth noting that there is some controversy regarding the length of MNs for corneal tissue. Some studies suggested that the length of MNs should be matched within the maximum injection depth (43–63 ​μm) corresponding to the human corneal epithelium (approximately 50 ​μm thick) without causing irreversible corneal stromal scarring [137,138]. However, in a separate study, MNs with a length of 600 ​μm and an insertion depth of approximately 150 ​μm did not cause significant inflammation or angiogenesis in animal tests [90]. Therefore, more detailed studies are still needed to explore the requirements of different tissues for the length and insertion depth of MNs.

**Table 2:** MNs for corneal regeneration.

Materials	Structure	Bioactive substances	Mechanisms	Ref.
Frozen glycerin	Arrays: 3 ​× ​3 Height: 400–440 ​μm Shape: pyramidal Strength: 00.3–0.4 ​N/neelde	*Bdellovibrio bacteriovorus*	Bacterial activity exceeded 80% after 14 ​d of storage.	[85]
Needle: outer MeHA shell and internal HA Base: HA	Arrays: 3 ​× ​3 Height: 500 ​μm Shape: pyramidal Strength: 0.4 ​N/neelde	Anti-angiogenic monoclonal antibody (DC101), diclofenac	Rapid dissolution of the outer layer of the needle tip, releasing >80% of the drug within 5 ​min; Slow release of the inner hydrogel layer (over 3 ​d).	[90]
Needle: silicon Base: PVA	Arrays: Height: 60 ​μm Shape: Circular Strength:	Bevacizumab	Painless and long-term sustained delivery of ocular drugs over the course of months.	[137]
PLGA, SU8 resin	Arrays: 1 needle Height: 150 ​μm Shape: Tapered Strength: 00.3–0.4 ​N/neelde	Antibiotics	The drug-tip is separated from base and released drugs after the hydrolysis of PLGA occurred in cornea.	[143]
Poly(D,l-lactide), HA	Arrays: 20 ​× ​20 Height: 390 ​μm Shape: pyramidal Strength:	Fluconazole	Penetrating the corneal epithelial layer reversibly; Increasing the residence time of drug in the conjunctival sac	[144]

**Fig. 7:**
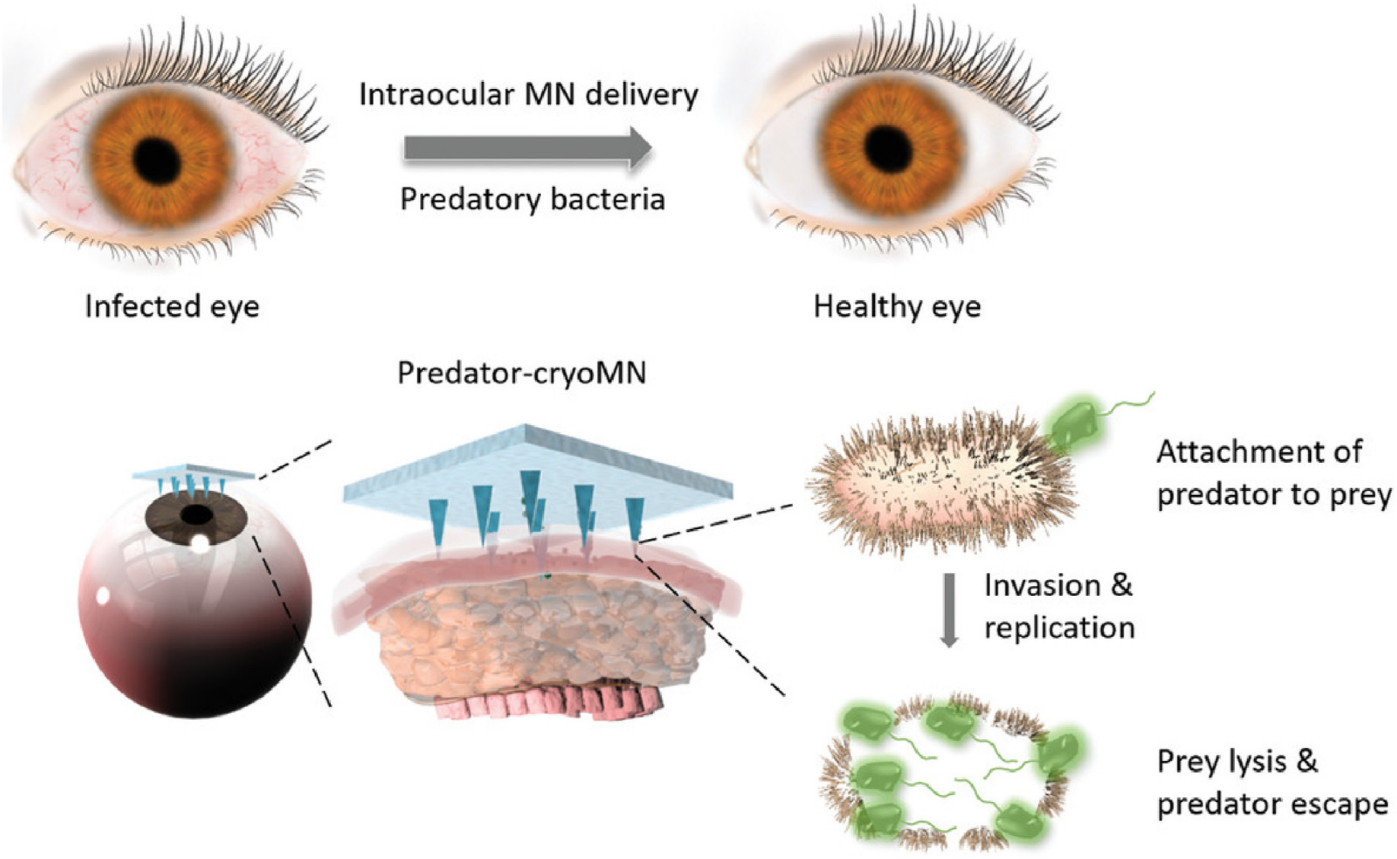
Illustration of cryoMNs for ocular delivery of predatory bacteria in treating eye infection (Reproduced with permission of Ref. [85]).

Keratitis caused by bacterial or fungal infections is one of the leading causes of corneal clouding and is the fourth leading cause of blindness worldwide [[139], [140], [141], [142]]. Antifungal drugs or antibiotic-loaded MNs have been shown to possess higher bioavailability and better therapeutic efficacy compared to eye drops [143,144]. To address bacterial drug resistance, Xu et al. prepared a novel frozen MNs by filling PDMS molds with glycerol/PBS/predatory *Bdellovibrio bacteriovorus* (*B. bacteriovorus*) bacterial suspensions and freezing them at −80 ​°C (as shown in Fig. 7) [85]. The frozen MNs could release *B. bacteriovorus* to remove gram-negative bacteria. This novel frozen MNs could provide an efficient delivery method for therapeutic organisms such as stem cells, which can adequately maintain their activity. However, such MNs need to be stored in a cryogenic environment before use, raising the cost of storage and transportation.

Alkali burns or inflammation can cause abnormal angiogenesis in corneal tissue, leading to vision loss. Chen et al. prepared a bilayer MN with biphasic drug release kinetics to treat corneal alkali burns [90]. The MN consisted with a methacrylated HA (MeHA) hydrogel shell containing anti-angiogenic monoclonal antibody (DC101) and HA core containing anti-inflammatory compound (diclofenac). The prepared MN could rapidly release diclofenac and continuously release DC101 to inhibit angiogenesis.

Although MNs have been shown to be a safe and effective delivery device for corneal repair, there is still some controversy regarding the needle length of MNs. In addition, the curvature of the cornea and the nature of the background-free support place certain demands on the way MNs are applied and the flexibility of the backing material. The available literature discusses less about the insertion efficiency of MNs for corneal regeneration, especially when the array number of MNs is large. Therefore, MNs for corneal tissue repair need to be designed in a more scientific manner. Although some studies designed a micro-needle pen with a spring to provide puncture force [77], this pen could only deliver a single MN. In addition, the angle of the pen was more difficult to control for different locations of the cornea.

### 3.3 MNs for endometrial repair

The endometrium is the epithelial layer on the surface of the uterine cavity and plays an important role in embryo implantation and pregnancy maintenance. Common events such as cervical dilatation/scraping and inflammation predispose to impaired endometrial regeneration, scar formation, fibrosis, and intrauterine adhesions (IUA) [[145], [146], [147]]. IUA is a gynecological condition in which scar tissue forms in the uterus, causing a range of symptoms, including recurrent miscarriages, amenorrhea, menorrhagia, cyclic pelvic pain, and even infertility [69,148,149]. The incidence of IUA has been reported to range from 6% to 30% in women who have undergone all types of abortions. In addition, the incidence of IUA can be as high as 25% in women who have undergone dilation and curettage after delivery [150,151]. Table 3 summarizes recent studies of MNs for endometria tissue regeneration. Typically, the length of MNs used for endometrial tissue repair falls within range of 500–1000 ​μm.

**Table 3:** MNs for endometrial regeneration.

Materials	Structure	Bioactive substances	Mechanisms	Ref.
GelMA	Arrays: Height: Shape: conical Strength: 0.3 ​N/needle	CeO_2_ nanozyme, stem cell	antioxidant CeO_2_ loaded base removed excessive ROS; Hydrogel needles improved stem cell activity, targeting efficiency and dwell time.	[152]
Arrowhead needles: GelMA; Base: bottom layer was N-acryloyl glycinamide (NAGA), top layer was PEGMA	Arrays: Height: 760 ​μm Shape: Arrowhead Strength: 0.2 ​N/needle	bFGF	The arrowhead needles allowed MNs steadily adhering to the tissues and released growth factors; Backing layer prevented tissue adhesions	[153]
GelMA,	Arrays: Height: 600 ​μm Shape: conical Strength: 0.2 ​N/needle	Lactoferrin, human endometrium-derived adventitial cells	Cells could rapidly form more biologically active three-dimensional cell spheres within the micropores surrounding the needles. Lactoferrin inhibited bacterial infections	[154]

**Fig. 8:**
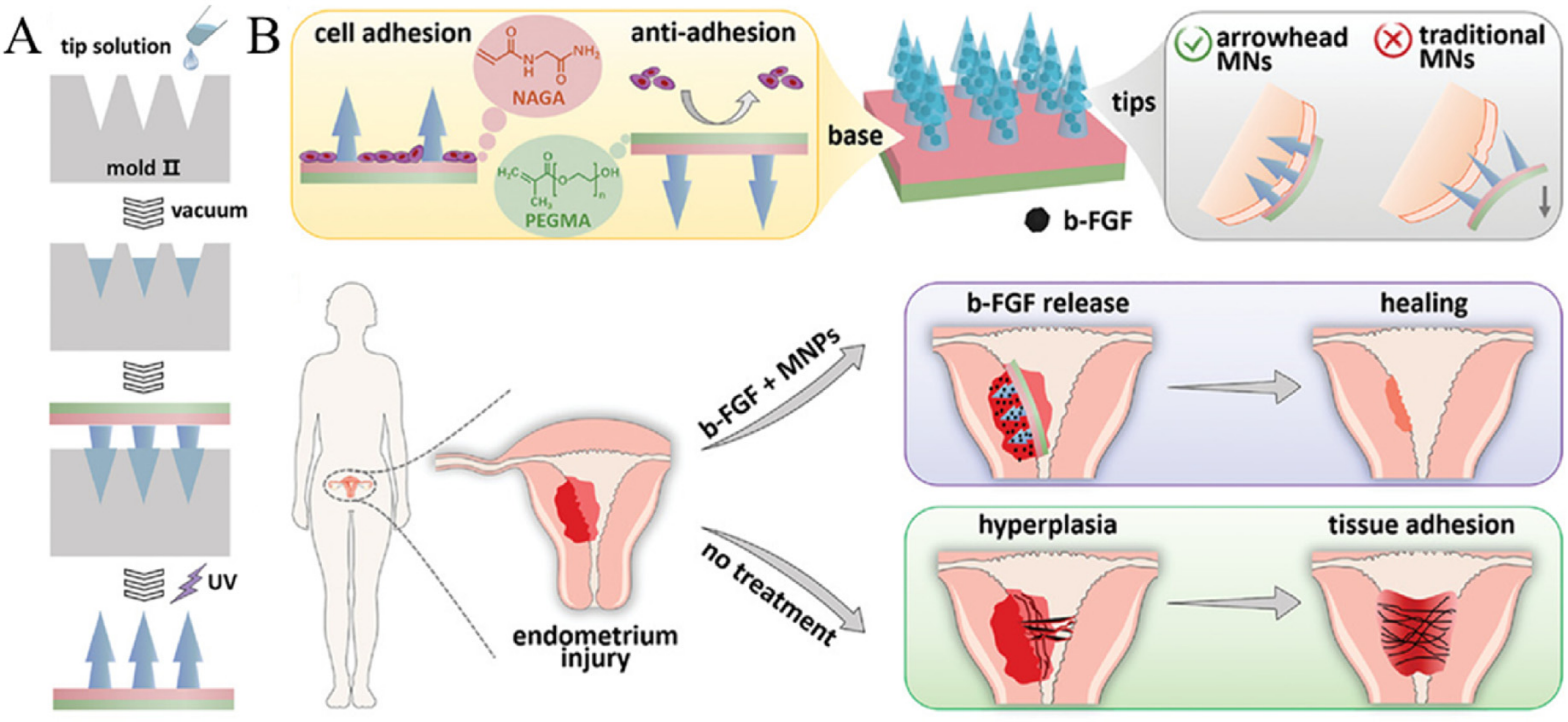
A) Preparation of arrowhead composite MNs. B) Schematic illustrations of the properties of the arrowhead composite MNs and their applications in endometrium repair and IUAs prevention (Reproduced with permission of Ref. [153]).

In order to scavenge ROS and improve the regenerative capacity of endometrium, Zhao et al. embedded antioxidant cerium oxide (CeO_2_) nanoenzymes and stem cells in the backing layer and tip of GelMA MNs, respectively [152]. The experimental results showed that the prepared MNs could promote the morphological reconstruction of damaged uterus, improve the pregnancy rate and restore the reproductive function. In addition, their group designed a new type of composite MNs with the stylized tip and anisotropic surface adhesion (as shown in Fig. 8) [153]. The tips of the MNs could form an interlock with the tissue, allowing the MNs to adhere stably to the tissue. And the tip was fabricated with cell-adhesive hydrogel containing growth factors, while its backing was an anti-adhesive polyethylene glycol methacrylate (PEGMA) hydrogel. Therefore, the prepared MNs could promote cell adhesion to repair damaged tissues while inhibiting tissue contact on the other surface to prevent undesirable adhesion.

In another study, Ding et al. prepared a layered GelMA hydrogel MNs containing lactoferrin (LF) with anti-inflammatory activity and antibacterial properties and human endometrium-derived epithelial cells (En-ADVs) [154]. The study designed a series of micropores on MNs in which EnADVs could be cultured as three-dimensional (3D) cell spheres. Compared to conventional monolayers or 3D dissociated cells, 3D cell spheroids exhibited enhanced maintenance of pluripotency, cell proliferation, cell migration, and promotion of angiogenesis.

Given their unique structure and good biocompatibility, MNs can effectively deliver active substances to achieve endometrial regeneration while acting as a physical barrier to prevent tissue adhesion. However, there are relatively few studies on MNs for the treatment of IUA, and large-scale animal experiments and clinical trials are still needed to verify their effectiveness. Future research in the field of endometrial damage repair by MNs may favor flexible combinations between multiple active substances (such as drugs, cells, growth factors and exosomes) and MNs, providing a wealth of options for the treatment of IUA. And it is important to consider the delivery system of MNs and the way they are inserted into the tissue when they are applied clinically to promote endometrial repair.

### 3.4 MNs for cardiac repair

Ischemic cardiovascular diseases such as MI are the leading cause of death worldwide. MI is a process of adverse cardiac remodeling triggered by apoptosis and necrosis of myocardial cells, activation of inflammatory response, degradation of extracellular matrix (ECM) and tissue fibrosis following acute or chronic blockage of coronary arteries [69,155,156]. There is still no effective treatment to promote cardiac regeneration [157]. Common clinical treatments mainly include drug therapy, heart transplantation and left ventricular assist device implantation [158]. However, due to the limited regenerative capacity of the heart, existing treatments can only temporarily maintain cardiac function and do not repair infarcted myocardial tissue [102]. There are also disadvantages such as heart size mismatch, vascular lesions, and shortage of supply [158]. Therefore, the development of scaffolds for cardiac tissue regeneration is expected to address these deficiencies and has important clinical implications. Recently, MNs have emerged as a promising tool with great versatility for delivering a variety of active substances to treat MI. Table 4 summarizes recent studies of MNs for cardiac tissue repair. And the length of MNs used for cardiac tissue repair is also 500–1000 ​μm, which is similar to skin and endometrium.

**Table 4:** MNs for cardiac repair.

Materials	Structure	Bioactive substances	Mechanisms	Ref.
PVA	Arrays: 20 ​× ​20 Height: 600 ​μm Shape: conical Strength: 2 ​N/needle	Cardiac stromal cells	Serving as the channels to allow for communication between the patch and the host myocardium, and transport nutrients and cytokines.	[159]
Needle: Elastin-like peptide Base: HA	Arrays:- Height: 600 ​μm Shape: conical Strength:	Mesenchymal stromal cell-secreted factors	The base was detached once MNs contacted the pleural fluid; Hydrogel tips in the myocardium allowed for continuous release of cytokines.	[160]
PVA	Arrays: 44.75 ​± ​1.28 needles Height: 850 ​μm Shape: conical Strength: Young's modulus of 12.28 ​± ​0.80 ​MPa	Adeno-associated virus	Achieving homogeneous distribution of adeno-associated virus delivery.	[161]
GelMA	Arrays:- Height:- Shape: pyramidal Strength:-	Carbon nanotube, cardiomyocytes	Adhering to the heart and released the encapsulated drugs; Ensuring the simultaneous contraction of cardiomyocytes distributed on the patch, made these cells to keep synergies with the heart in *vivo*.	[162]
PVA	Arrays: 11 ​× ​11 Height: 600 ​μm Shape: conical Strength: puncture force of 4.7 ​N	VEGF, graphene oxide	Folded MNs could be implanted minimally invasively and could pierce the myocardium and wrap the heart when fully expanded under NIR irradiation; Sustained release of VEGF.	[163]
GelMA	Arrays: 385 needles Height: 570 ​μm Shape: conical Strength: 0.4 ​N/needle	Galunisertib	Sustainably released galunisertib for more than 2 weeks and provided mechanical support.	[164]

**Fig. 9:**
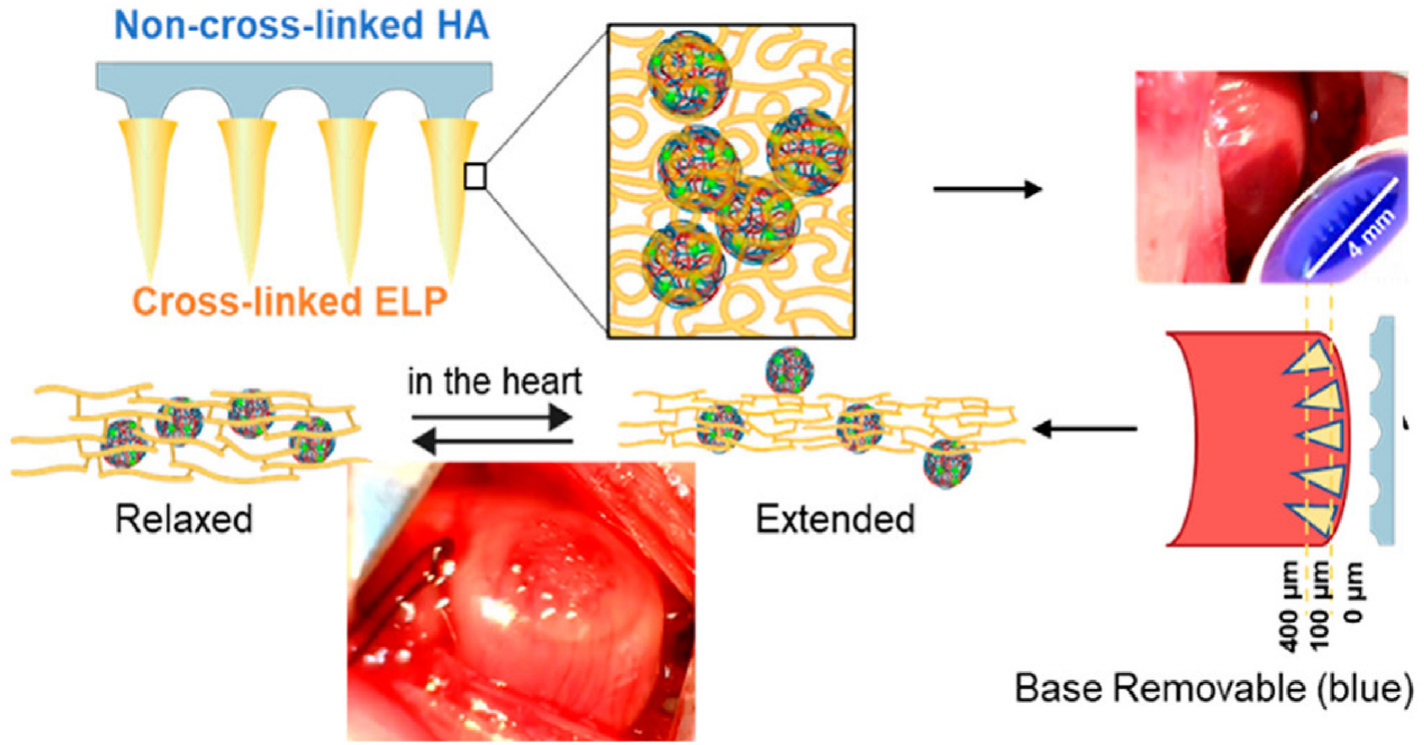
MSCF loaded detachable MNs for cardiac repair (Reproduced with permission of Ref. [160]).

MNs can act as a channel between the patch and myocardial tissue. Prof. Cheng's group prepared PVA MNs carrying cardiac stromal cells [159]. The MNs could aspirate interstitial fluid from the heart to provide nutrients to the cells, while releasing paracrine factors to repair the heart. The results of animal experiments demonstrated that the prepared MNs could promote MI healing by promoting angiogenesis, reducing scar size and enhancing cardiac function. Subsequently, to avoid the cardiac burden caused by the sutures of the MNs, they designed a detachable MNs (as shown in Fig. 9) [160]. The tip of this MNs consisted of PLGA nanoparticles loaded with mesenchymal stromal cell secretory factor (MSCF) and elastin-like peptide hydrogel, and the basing of the MNs was dissolvable HA. Once in contact with tissue fluid, the backing would fall off quickly, so no sutures were required. Instead, the tips could be firmly inserted into the infarcted myocardium and release MSCF to reduce myocardial apoptosis, restore myocardial volume, and decrease myocardial fibrosis. Similarly, other active substances including adeno-associated virus (AAV) and VEGF, IL-10 were also encapsulated into MNs for MI treatment [[161], [162], [163]].

On the other hand, MNs can also act as myocardial patches, providing mechanical support for heart to reduce myocardial stress and inhibit unfavorable ventricular remodeling [164]. However, MNs are currently provided through invasive open-heart surgery, which increases the risk of infection and cardiac burden on patients. Inspired by the venomous stinger of honeybees, Prof. Zhu's group prepared bionic MNs with a barbed structure and embedded the MNs in an elastomeric film [96]. The prepared MNs could firmly fix to the beating heart, reduce the wall stress in the infarcted area and maintain the function and morphology of the left ventricle. In addition, the MNs could be minimally invasively implanted into the beating porcine heart using thoracoscopy in less than 10 ​min without sutures and adhesives. However, the process was studied in the normal heart.

In conclusion, MNs can promote MI repair by delivering active substances to infarcted myocardial tissue or providing mechanical support. MNs allow better integration of biomaterials and infarcted myocardial tissue than conventional patches. Tissue-adherent MNs can be delivered in a minimally invasive manner, thus avoiding suturing and open-heart surgery. However, most of the animal models are acute MI caused by ligation of the left anterior descending branch. In the future, more models are still needed to further validate the therapeutic effect of MNs. On the other hand, whether MNs can damage the normal tissue surrounding the infarcted myocardial tissue, destroy capillaries or cause inflammation still needs to be rigorously verified.

### 3.5 Others

In addition to skin, cornea, endometrium, and myocardium, MNs have been also widely explored as controlled local delivery platform of active substances for the repair of damaged tissues such as spinal cord injury (SCI), stroke, and periodontal diseases. And relevant studies are summarized in Table 5.

**Table 5:** MNs for other tissues regeneration.

Tissue	Materials	Structure	Bioactive substances	Mechanisms	Ref.
Spinal cord	Silicon coated with gold and polypyrrole	Arrays: 10 ​× ​10 Height: 500 ​μm Shape: conical Strength:-	Dexamethasone phosphate	Transdural and electronically controlled delivery of drugs to the intrathecal space.	[170]
	GelMA	Arrays: 45 needles Height: 600 ​μm Shape: conical Strength: <1.5 ​N/needle	Mesenchymal stem cell	Maintaining the activity of cells for more than 7 ​d; Achieving sustained delivery of exosome.	[171]
	GelMA	Arrays: 20 ​× ​20 Height: 300 ​μm Shape: conical Strength: Young's modulus of 500 ​kPa	Mesenchymal stem cell-derived exosome	Improving the exosome retention rate, achieved sustained release over 6 ​d.	[172]
Periodontal tissue	GelMA and gelatin	Arrays: 11 ​× ​11 Height: 600 ​μm Shape: conical Strength: about 4 ​N	Tetracycline, IL-4 and TGF-β	Quickly dissolvable gelatin base for burst release of antibiotic; Antibiotic loaded nanoparticles and cytokine loaded silica microparticles in needles for sustained release.	[176]
Oral mucosal	HA	Arrays: 10 ​× ​10 Height: 350 ​μm Shape: conical Strength: penetrate the oral mucosa	dexamethasone acetate, vitamin C and tetracaine hydrochloride	Fully released the encapsulated drug within 10 ​s.	[177]
	HA	Arrays: 15 ​× ​15 Height: 700 ​μm Shape: conical Strength:	Betamethasone	Fully released the encapsulated drug within 3 ​min.	[178]
Brain	GelMA	Arrays: 10 ​× ​10 Height: 600 ​μm Shape: conical Strength: >0.07 ​N/needle	Adeno-associated virus	For sustained and controlled delivery of adeno-associated virus (AAV) expressing human VEGF (AAV-VEGF) that achieves homogenous distribution and high transfection efficiency in ischemic brains.	[182]
Skeletal muscle	PLGA needles coated tungsten	Arrays: 5 ​× ​5 Height: 900 ​μm Shape: pyramidal Strength: modulus of 77 ​MPa	Aspirin and ibuprofen	The base was combined with wireless power transmission system; Transmission of periodic electrical stimulation to regulate cell behavior and tissue regeneration, release anti-inflammatory drug.	45
Cartilage	–	–	cellular microspheroids	Microspheroids on MNs were cultured to permit fusion into a tissue construct.	[187]
Liver	GelMA	Arrays: 11 ​× ​11 Height: 577 ​μm Shape: conical Strength: 0.054 ​N/needle	silicate nanoplatelets	The needle-shaped structure increases the contact area with blood. Adhering to the wound through an interlocking mechanism.	[190]
	HA	Arrays: Height: 750 ​μm Shape: pyramidal Strength: ∼0.2 ​N/needle	Tranexamic acid	Peripheral MN for minimally and site-selectively invasive hemostatic drug delivery. The released drugs were transported from adjacent tissue to the target site through blood flow in capillaries.	[191]
Blood vessel	208CTH-F	Arrays: Height: 200 ​μm Shape: octagonal based cones Strength:	paclitaxel	MNs covered on the surface of drug-eluting balloons. Balloon expansion allowed MNs to penetrate the inner layer of the vessel wall and released the drug on the surface.	[193]
	PLGA	Arrays: 3 ​× ​3 Height: 650 ​μm Shape: conical Strength:	Rhodamine B, paclitaxel	The combination with the cuff-shaped device allowed MNs to be applied to the outer surface of the vessel; Drug released by MNs were uniformly distributed in the vascular tunica media over 2 weeks.	[194]
	PLGA	Arrays: 4 ​× ​4 Height: 480, 640 and 780 ​μm Shape: pyramidal Strength: Young's moduli of 29.7 ​MPa	rhodamine B, sirolimus	The MNs are mounted on a flexible PLGA mesh that released drug to the vessel wall and minimized the mechanical stresses applied to the vessel.	[195]
	Needle: PCL, PLGA, lauric acid Base: Curable resin PCLMA	Arrays: Height: 500 ​μm Shape: conical Strength: >0.2 ​N/neelde	paclitaxel	MNs were mounted on the surface of the balloon; After irradiation by a circular NIR laser inside the catheter, the drug-carrying tip was dislodged and embedded in the vascular system and gradually released the drug for more than six months.	[196]

#### 3.5.1 Spinal cord

**Fig. 10:**
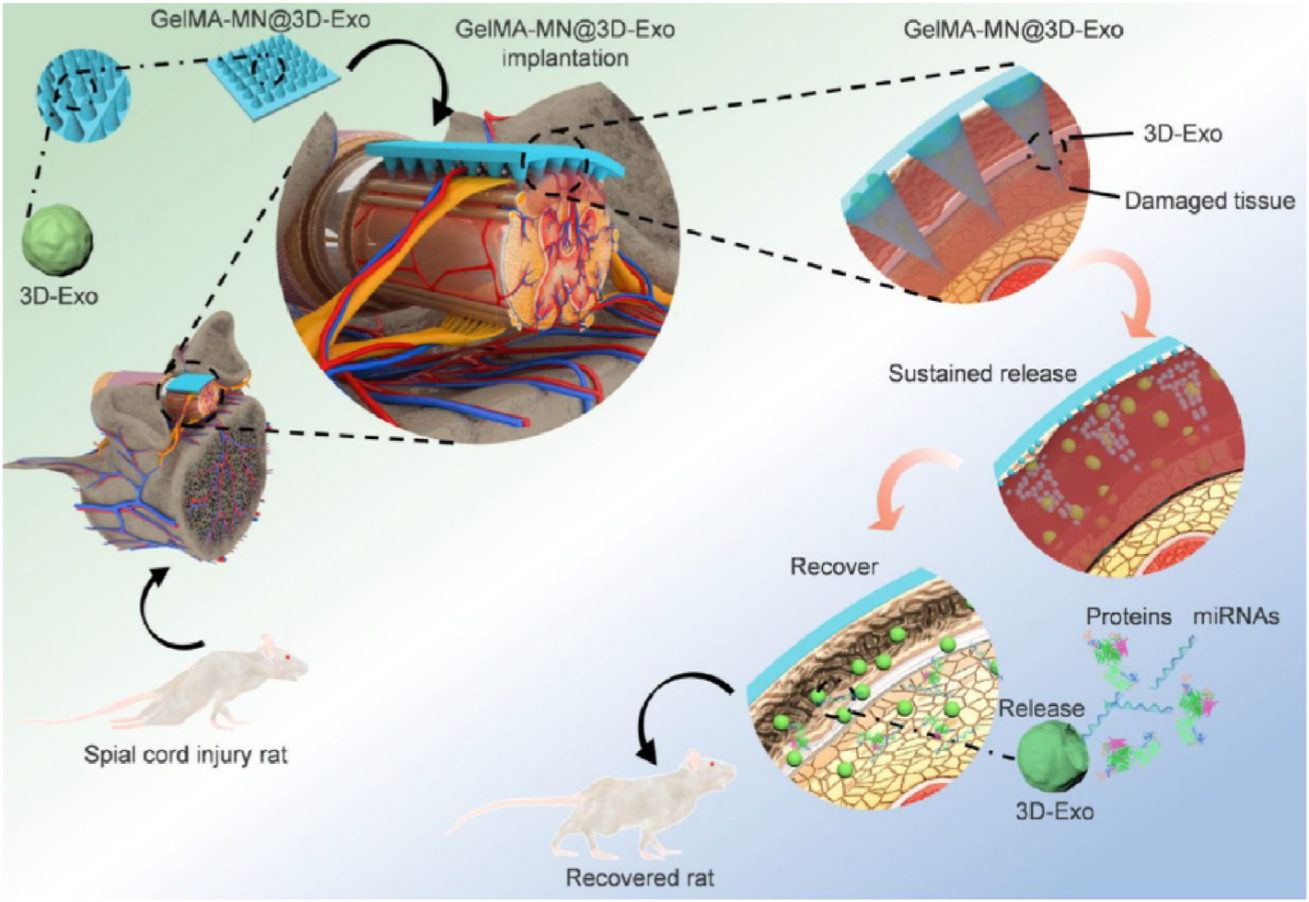
3D-Exo loaded MNs for SCI Repair (Reproduced with permission of Ref. [172]).

SCI is one of the most serious neurodegenerative diseases in modern society, causing sensory impairment and/or paraplegia in patients [165,166]. SCI can be caused by a variety of reasons, including traffic accidents, industrial accidents, and sports injuries [[167], [168], [169]]. Improving neurological recovery in SCI patients has remained a challenge over the past few decades. MNs has been shown to deliver the active substance more effectively without damaging nearby spinal tissue than traditional local repeat injections [170,171]. As illustrated in Fig. 10, Xin et al. cultured mesenchymal stem cells in a hydrogel and obtained exosomes (3D-Exo) containing a large number of proteins and miRNAs involved in local microenvironment regulation [172]. And they implanted 3D-Exo loaded GelMA hydrogel MNs into a rat SCI model. The Young's modulus of MN@3D-Exo determined by atomic force microscopy (AFM) was 500 ​kPa, which matched with normal spinal cord (200−600 ​kPa). The *in vivo* results showed that local implantation of MNs significantly improved the retention rate of 3D-Exo, achieved slow release of active ingredients, and promoted the recovery of neural function. It is important to note that the mechanical properties of MNs should match those of the spinal cord in order to avoid causing tissue damage. In addition, the optimal time window for implantation of MNs remains to be further investigated.

#### 3.5.2 Oral tissues

Periodontitis is a chronic inflammatory disease that leads to progressive destruction of periodontal tissue (including alveolar bone, periodontal ligament and root cementum) and is the leading cause of tooth loss in adults [173]. For various reasons (such as oral hygiene, defect size, infection, and many others), injured periodontal tissue may not be able to repair itself through wound healing and tissue regeneration [174,175]. Therefore, advanced regenerative therapy need to be developed to restore the original structure and function of periodontal tissue. To clear microbial infections and modulate immune responses, Li et al. developed a biodegradable MN that could be implanted into gingival tissue in a painless and suture free manner [176]. The prepared MN consisted of a fast-dissolving adhesive backing for immediate release of tetracycline and biodegradable GelMA hydrogel tips containing tetracycline loaded PLGA nanoparticles and cytokine loaded silica particles. The tips could continuously release antibiotics to completely inhibit bacterial growth, and release IL-4 and TGF-b to induce polarization of anti-inflammatory macrophages and formation of regulatory T cells. In addition, MNs have also been shown to painlessly penetrate the tongue abdomen mucosa and release drugs to treat oral ulcers, another common oral inflammatory disease [177]. Wang et al. prepared dissolving HA MNs containing betamethasone phosphate sodium (BSP) and betamethasone dipropionate (BDP) [178]. HA MNs could release BSP and BDP to the base of the ulcer (to a depth of more than 200 ​μm) in less than 3 ​min. The experimental results demonstrated that MNs could improve the comfort and efficacy of oral ulcer treatment compared to commercially available topical injections and creams.

#### 3.5.3 Brain

Stroke is an ischemic or hemorrhagic disease mainly occurring in the microvascular system of the brain, resulting in cerebrovascular damage and irreversible damage to neurological function. And stroke is one of the major causes of death and severe disability worldwide [179,180]. Intravenous thrombolysis to restore blood flow to the ischemic site is the main treatment for ischemic stroke. However, thrombolysis can also lead to a sharp increase in oxygen concentration at the injured site after ischemia-reperfusion, which may aggravate brain injury [181]. In order to promote angiogenesis and functional reconstruction in infarction brain tissue, Wang's group innovatively proposed a GelMA MN for continuous and controlled local delivery of human VEGF expression adeno-associated virus (AAV) [182]. Ischemic stroke models were established in adult rats, and an AAV-VEGF-loaded MNs cortex was inserted into the ischemic core and penumbra of these rats one day after the ischemic attack. The *in vivo* results showed that MNs did not cause significant inflammatory response, and achieved successful transfection and uniform distribution in the cerebral cortex within three weeks after surgery. And the prepared MNs increased VEGF expression and enhanced functional angiogenesis and neurogenesis.

#### 3.5.4 Muscle

Electrical stimulation is a physical and convenient treatment that has been widely used to promote cell proliferation and tissue regeneration [183,184]. In a pioneering study, Yu et al. prepared conductive MN electrodes by hot pressing PLGA MNs loaded with aspirin and ibuprofen onto Mg foils and then depositing W or Mg onto MN [45]. When combined with radio frequency (RF) based wireless power transmission systems, the MNs could be externally controlled and programmed to generate target waveforms for target tissues. Drugs were continuously released from the MNs during the dissolution of the device to prevent inflammation. In a rat model of muscle injury, the device not only shortened the duration of muscle regeneration, but also reduced the inflammatory response.

#### 3.5.5 Cartilage

Cartilage defects due to osteoarthritis are a serious tissue lesion for which effective treatment is still lacking [185]. As a minimally invasive drug delivery device, MNs are often used for transdermal delivery of anti-inflammatory drugs or immunosuppressants to treat arthritis and promote cartilage repair [186]. D'Lima et al. assembled stem cell microspheroids of 500 ​μm diameter individually on MNs in a predetermined arrangement to form a construct of desired size and shape [187]. Microspheroids on MNs were cultured for 4–5 days to permit fusion into a tissue construct. This tissue construct showed good pro-chondral repair ability in the repair of *ex vivo* osteoarthritic human cartilage and *in vivo* rabbit osteochondral defects.

#### 3.5.6 Liver

Limited by the poor effectiveness of traditional clinical hemostatic techniques such as suturing, uncontrolled or excessive visceral bleeding caused by acute trauma is a common and serious medical problem, often resulting in patient death [188,189]. In section 2.4, we have discussed the ability of a biomimetic pagoda-like MNs to immediately repair acute tissue injuries such as hepatic hemorrhage, splenic hemorrhage, and renal hemorrhage by physical interlocking [98]. In addition, recent studies have found that the needle-like structure of MNs could increase the contact area of the material with blood, thus further accelerating liver repair and hemostasis [190]. A silicate nanosheet-loaded GelMA MNs exhibited exciting hemostatic effects and could reduce clotting time by 89%, exceeding most commercially available solid-based hemostatic agents. And in a rat liver hemorrhage model, the MNs reduced bleeding by approximately 92% compared to the untreated injury group. Furthermore, to avoid secondary damage to the liver injury site, a peripheral MNs was designed by Lim et al. [191] The drug released by this MNs was transported from adjacent tissues to the target site via blood flow in the capillaries.

#### 3.5.7 Blood vessel

The microscopic thickness of the outer and inner vessel membranes results in low bioavailability of traditional intravascular drug delivery methods such as balloons and stents [51]. The high rate of restenosis and neointima formation has prompted investigators to diligently pursue new peritascular or intravascular delivery methods, and MNs offer a potential alternative [35,192]. Unlike traditional MNs used for other tissue repair, MNs used for vascular repair usually require special structural design, combined with flexible substrates or balloons, to ensure that MNs can penetrate the vascular wall without penetrating [193,194]. Due to the highly curved or complex vascular structures, which have repetitive pulsatile movements, base of the MNs for vascular repair needs to be more flexible so that they can better fit the vascular wall and avoid vascular damage. For example, a type of PLGA MN mounted on the surface of a PLGA fabric mesh could wrap around the outer vascular wall to enhance drug delivery efficiency and minimize the mechanical stresses applied to the vessel [195]. Most of the earliest MNs used for vascular repair were coated microneedles with limited drug loading and treatment period. Wang et al. equipped the tip detachable MN on the balloon surface to achieve longer-lasting drug release for more than half a year [196]. The drug loaded tips of the MN consisted of the biodegradable material PLGA/PCL and lauric acid with low melting point. The annular laser fiber in the inner axis of the catheter emitted NIR to heat and melt the lauric acid. After the molten lauric acid was converted to a solid state, the bond between the MN tip and the base was weakened. And the drug loaded tip could be separated from the base by pulling the balloon catheter by hand.

As a novel *in situ* delivery platform, MNs have demonstrated excellent ability to promote tissue regeneration in animal models of SCI, stroke, periodontitis and muscle injury. However, due to the lack of sufficient experimental data and clinical research support, the therapeutic effect of MNs needs to be further verified. At the same time, MNs are expected to be applied in more other tissues, bringing new opportunities for healing damaged tissues.

## 4 Future perspectives and conclusions

After more than ten years of exploration, a variety of multi-functional MNs represented by dissolving or hydrogel MNs have been developed for regeneration of various tissues, including skin, cornea, endometrium, myocardium, muscle, spinal cord, oral tissues and blood vessel. Among these, difficult-to-heal wound repair such as diabetic ulcers and bacterially infected wounds are currently the most extensively researched areas for MNs. In general, MNs offer several unique advantages over traditional scaffolds such as films, sponges and hydrogels.

1. The micro-needle structure provides strong penetration to help biomaterials pierce the necrotic tissues, biofilms, vascular wall, corneal epithelial layer and other tissue barrier, thereby improving the drug bioavailability.
2. Flexible combinations of materials (dissolvable or degradable, and hydrogels) and structures (needle tips and bases) allow control of drug release rates, and drug spatial distribution on the tissues surface or deep layer, and damaged area or adjacent normal tissue.
3. Regulating cell behavior by generating physical stimuli or providing engagement/adhesion for rapid wound closure.
4. With their flexible preparation methods (solvent casting, 3D printing and drawing lithography et al.), material properties (strength, flexibility and melting point et al.) and geometry, MNs can be delivered to the target tissue site by thumb press, vacuum pump, spring-loaded pen, balloon and catheter, etc., thus meeting the requirements of clinical applications.

Most of the current studies are using MNs as a device for drug delivery to improve delivery efficiency. But studies on the effects of the physical properties of MNs, including geometry, needle height, tip radius, needle density and material, on the tissue repair process are still lacking. As a drug delivery platform, the problem of low drug loading rate of MNs is still not well solved, therefore MNs require careful research on efficient drug delivery methods, intelligent drug release mechanism and pharmacokinetics. In addition, the *in vivo* tissue repair performance of MNs was mainly evaluated in small animals such as mice, rats and rabbits. Due to their anatomical, physiological and biomechanical structure, they differ from humans. Therefore, human volunteers will need to be recruited in the future to test the therapeutic effects of MNs.

Although MNs show promising potential in the field of tissue repair, unfortunately, most of the products currently on the market are for cosmetic and vaccination purposes. In addition, a search of the U.S. clinical trials database (clinicaltrials.gov) using the keywords ‘(microneedle) OR (microneedling) OR (micro needle) OR (microneedles)’ showed that more than 3/4 of the clinical trials are hollow or solid MNs in vaccination, dermatology, pain management, etc. And no clinical trials have been found on the use of MNs for ISTR. MNs used for ISTR have the ability to interact with and alter the microenvironment *in vivo*, resulting in the need for regulatory approval of all aspects of safety and performance (including host tissue acceptability, effects on gene expression and signaling, effects on local microenvironments, and safety or therapeutic efficacy of degradation products). In addition, MNs have been relatively poorly studied in tissues other than difficult-to-heal wounds. As a result, more verification and validation tests for MNs used in ISTR require more effort and resources.

## Declaration of competing interest

The authors declare that they have no known competing financial interests or personal relationships that could have appeared to influence the work reported in this paper.

## Data Availability

No data was used for the research described in the article.
